# The Book World of Medicine and Science

**Published:** 1898-03-26

**Authors:** 


### THE BOOK WORLD OF MEDICINE AND SCIENCE.

Pocket Formulary for the Treatment of Disease in Children. By Ludwig Freyberger, M.D., M.R.C.P., Clinical Assistant Hospital for Sick Children, Great Ormond Street; Curator of the Museum, Pathologist and Registrar Great Northern Central Hospital. (London: The Rebman Publishing Company. 1898. Price 6s. 6d. net.)

This is but a little book, but it is good, and contains a very large amount of information of daily utility and importance. The main part of it is devoted to an alphabetical list of the drugs used in the treatment of diseases of children, giving under each heading a brief account of their properties, uses, therapeutics, incompatibles, dose, correction of their taste, examples of formulæ, their antagonists and antidotes. Of course, the greater number of the preparations dealt with are to be found in the British Pharmacopœia, but besides these there are a considerable number of drugs which, although not official, are more or less frequently employed, and are mentioned in text books and in current medical literature. At the end of the book is a therapeutic index, which will no doubt be useful in sometimes jogging a memory which is tending to get in a groove. We doubt, however, whether such an index is likely to be of any real value. For example, let us take a few of the diseases, the names of which are followed by but one drug: "Angina pectoris, quebracho tinctura"; "Influenza, phenazonum"; "Intussusception, opii tinctura"; "Tenesmus, belladonnæ extractum"; "Tic douloureux, butyl chloral hydras." There is a sweet simplicity about this which, we think, hardly does justice to Dr. Freyberger's acumen. The real utility of the book, however, lies in its major part, especially in the formulæ and in the memoranda as to dosage.

Guide to Bath, Bristol, &c. (London: A. and C. Black. 1897; pp. 87. Maps. Price 1s.)

This is a sixth edition of Black's well-known and reliable guide. For decades past our British Naamans have neglected the Abanas and Pharpars of their own country, and sought health at the continental spas. Lately, however, fashion has in some degree changed. The only respect probably in which Bath as a health resort is inferior to continental resorts is in its proximity to London and the impossibility of escape from the fusilade of letters and telegrams which make rest impossible. Visitors to Bath, seeking either health or pleasure, should certainly read this guide, which is, so far as our knowledge goes, perfectly reliable. A considerable amount of space is devoted also to an account of Bristol and Clifton, and many excursions from both towns to places of interest in the district are described.

Elements of Latin for Students of Pharmacy and Medicine. By Geo. D. Crothers, A.M., M.D., and Hiram H. Bice, A.M. Pp. 241. (Philadelphia. The F. A. Davis Co. 1898.)

This is a delightful book. The authors, in their preface, state that "it is designed to present in a brief compass those principles of Latin etymology and construction which are essential to an intelligent use of the terminology of pharmacy and medicine." But the chief characteristic of American humour is the gravity with which extravagances are related, and we cannot acquit Drs. Crothers and Hiram H. Bice of concocting an excellent, though elaborate *jeu d'esprit*. The book is cast in the form of the ordinary Latin grammar, and declensions and conjugations are set forth with some show of form. But the "Exercises" are conceived and elaborated in the best Ollendorfian vein. We cull a few examples:

"116.—The wife of the old man buys a bottle of strong solution of ammonia. The son of the old husbandman has major epilepsy. The benevolent physician prescribes for the unfortunate youth bromide of potash."

"118.—The cheerful physician soothes the sick maiden with an anecdote."

But the quaintest of all is

"Exercise 131.—Puer parvus fructus viridis edit et——." The hiatus is the authors', not ours. The exercises are elucidated by notes. Thus we find in one exercise—"Nantes veteres spiritum frumenti ninium bibunt." And in the notes —"Spiritus frumenti=Whisky, an alcoholic liquor distilled from grain." A list of phrases suitable for use in prescribing are given, amongst them: "Ne fradas sine numme—Deliver not without money." And in a table of weights and measures we find allusions to "fluidounces" and "fluidrachms"! The book concludes with a list of "Anatomical Proper Names and their Origin," such as:—

"*Achilles*: Grecian hero—Tendo Achilles."

"*Adamus*: The first man—Pomum Adami."

"*Bartholin*: Danish physician—Glands of Bartholin."

So delicious a collocation could not be the result of anything but humorous design. Speaking seriously, we are quite at a loss to conceive how this extraordinary farrago of dog-Latin, anatomy, and pharmacy came to be strung together, or what purpose it could serve."

Outlines of Rural Hygiene. By Harvey B. Bashore, M.D. Illustrated. Pp. 77. Price 75 cents. (Philadelphia: The F. A. Davis Co. 1897.)

This little volume, written for "physicians, students, and sanitarians," is chiefly remarkable for its earnest and able advocacy of the "earth to earth" system of disposal of excreta and waste. In this, as in its title, it is reminiscent of Dr. Poore's well-known book. A chief point, however, in Dr. Poore's rural economy to which the author takes exception is the provision of a water supply from shallow wells. There is no doubt that the weakness of the "earth" system is in the opportunity it affords for contamination of shallow wells, although Dr. Poore's famous well at Andover yields a good supply of bacteriologically pure water. Recent researches, especially those of Dr. Martin, on the vitality in earth of B. typhosus, suggest that specific pathogenic organisms may penetrate and exist where others perish. Dr. Bashore has written an excellent and interesting book.

